# Mouse IgG3 binding to macrophage-like cells is prevented by deglycosylation of the antibody or by Accutase treatment of the cells

**DOI:** 10.1038/s41598-021-89705-3

**Published:** 2021-05-13

**Authors:** Alicja Karabasz, Monika Bzowska, Joanna Bereta, Maria Czarnek, Maja Sochalska, Tomasz Klaus

**Affiliations:** 1 Department of Cell Biochemistry, Faculty of Biochemistry, Biophysics and Biotechnology, Jagiellonian University in Kraków, 7 Gronostajowa, 30-387 Kraków, Poland; 2 Department of Microbiology, Faculty of Biochemistry, Biophysics and Biotechnology, Jagiellonian University in Kraków, 7 Gronostajowa, 30-387 Kraków, Poland; 3 Present Address: Pure Biologics Inc., 11 Duńska, 54-427 Wrocław, Poland

**Keywords:** Integrins, Humoral immunity, Cellular immunity, Biochemistry, Flow cytometry, Cell culture, Antibody isolation and purification, Phase-contrast microscopy, Antimicrobial responses, Immunology, Monocytes and macrophages, Glycosylation

## Abstract

The binding of mouse IgG3 to Fcγ receptors (FcγR) and the existence of a mouse IgG3-specific receptor have been discussed for 40 years. Recently, integrin beta-1 (ITGB1) was proposed to be a part of an IgG3 receptor involved in the phagocytosis of IgG3-coated pathogens. We investigated the interaction of mouse IgG3 with macrophage-like J774A.1 and P388D1 cells. The existence of an IgG3-specific receptor was verified using flow cytometry and a rosetting assay, in which erythrocytes clustered around the macrophage-like cells coated with an erythrocyte-specific IgG3. Our findings confirmed that receptors binding antigen-free IgG3 are present on J774A.1 and P388D1 cells. We demonstrated for the first time that the removal of N-glycans from IgG3 completely abolished its binding to the cells. Moreover, we discovered that the cells treated with Accutase did not bind IgG3, indicating that IgG3-specific receptors are substrates of this enzyme. The results of antibody-mediated blocking of putative IgG3 receptors suggested that apart from previously proposed ITGB1, FcγRII, FcγRIII, also additional, still unknown, receptor is involved in IgG3 binding. These findings indicate that there is a complex network of glycan-dependent interactions between mouse IgG3 and the surface of effector immune cells.

## Introduction

Two of the major effector functions of IgGs, pathogen phagocytosis and antibody-dependent cell-mediated cytotoxicity (ADCC), are executed via Fc-binding receptors (FcγRs). FcγRs are divided into high-affinity receptors, which bind free IgGs, and low-affinity receptors, which bind IgGs complexed with a polyvalent antigen. Although four FcγRs have been described in mice, none of them interacts with free mouse IgG3 molecules with high affinity^1^.

Mouse IgG3 (mIgG3) is a unique antibody subclass that is highly protective against pathogens with a capsule, e.g., *Neisseria meningitidis* or *Bacillus anthracis*. The mechanism of mIgG3 efficacy is unknown^2,3^. However, it was shown that on the surface of mouse immune cells, including macrophages, there is an unidentified receptor that binds mIgG3^4–6^. This unknown receptor mediates the recognition of pathogens opsonized with mIgG3s^5,6^.

In 1981, the existence of a mIgG3-specific receptor was proposed for the first time by Diamond and Yelton, who described a clone of the J774A.1 macrophage-like cell line that had lost the ability to bind mIgG3 while retaining the ability to bind to other IgG subclasses^4^.

Years later, Gavin et al. discovered that mouse FcγRI interacts with IgG3 with low affinity^7^. FcγRI molecules bind mIgG3s that opsonize a multivalent antigen. The complexing of many FcγRI molecules with mIgG3s is required to elicit the response of effector immune cells. Surface plasmon resonance (SPR) analysis of the interaction between mIgG3 and FcγRI showed that FcγRI does not bind to free IgG3s^8^. Flow cytometry analysis confirmed that the affinity of IgG3 to FcγRI is low^7^.

In 2010, Casadevall's group published a paper on the differences in the induction of *Cryptococcus neoformans* phagocytosis by pathogen-specific-mIgG3 and mIgG1^6^. In contrast to mIgG1, mIgG3 induced phagocytosis even in macrophages that did not express any known FcγRs (including FcγRI). Thus, this result confirmed the existence of a receptor specific to mIgG3. The authors also showed that there are approximately 10,000 molecules binding mIgG3 with nanomolar affinity on the surface of peritoneal macrophages^6^.

This same group tried to identify the mIgG3-specific receptor using a short hairpin RNA (shRNA) library. The authors identified integrin β1 (ITGB1) as a part of mIgG3 receptor complex involved in phagocytosis^5^. However, direct evidence of an interaction between mIgG3 and ITGB1 was not shown.

We investigated mIgG3 binding to macrophage-like J774A.1 and P388D1 cells and confirmed the existence of a receptor that binds antigen-free mIgG3. We showed for the first time that the binding of mIgG3 to the cells depends on N-glycans on the antibody. We also discovered that the receptor is susceptible to Accutase treatment, while trypsin does not affect mIgG3 binding to the cells. Consistent with reports published by other researchers, we confirmed the involvement of ITGB1, FcγRII, and FcγRIII in mIgG3 binding. However, contrary to Hawk et al.^5^, we did not observe a divalent ion-dependency of the interaction between IgG3 and the cells.

These findings of this study provide an essential perspective for the further search for mIgG3-specific receptors. We also showed how antibody quality and cell preparation might affect the results of experiments exploring the mIgG3 interaction with immune cells.

## Methods

### M18 IgG3 and its derivatives

Mouse IgG3 M18 was produced by a hybridoma clone described previously^9^. The antibody is specific to antigen B of the ABO blood group system. M18 was purified using CaptureSelect LC-kappa (mur) Affinity Matrix (Thermo Fisher) according to the instructions of the supplier. Glycine–HCl (100 mM, pH 2.0) was used for elution. Eluted fractions were immediately neutralized with 2 M TRIS and dialyzed into phosphate buffered saline (PBS). The antibody was stored at a concentration below 1 mg/mL to prevent its precipitation. M18 was deglycosylated under native condition using PNGase F (Promega) according to the manufacturer's protocol. Deglycosylation was confirmed by a band shift to a lower molecular mass in SDS-PAGE. M18 and its deglycosylated variant were conjugated with a fluorescent label using the DyLight 488 Microscale Antibody Labeling Kit (Thermo Fisher). Efficacy of antibody conjugation with the fluorescent label was analyzed using a fluorimeter. Fluorescence intensity of equimolar solutions of the labeled molecules was consistent between different batches and between antibody variants. Table 1 summarizes the names of the antibody derivatives used in the work.

**Table 1:** IgG3 and its derivatives used in experiments.

Name	Description
M18	Native anti-B antibody
degM18	Deglycosylated anti-B antibody
M18-488	Glycosylated anti-B antibody labeled with DyLight 488 fluorophore
degM18-488	Deglycosylated anti-B antibody labeled with DyLight 488 fluorophore

### Cell lines and culture conditions

Two mouse monocyte/macrophage-like cell lines: P388D1 (ATCC, CCL-46) and J774A.1 (ATCC, TIB-67) were used. The cells were cultured at 37 °C, 5% CO_2_ in DMEM (4.5 g glucose/l, Thermo Fisher) supplemented with 5% FBS (US origin, Thermo Fisher). The cells were subcultured by sloughing with a transfer pipet. The estrogen-regulated cell line of mouse neutrophil progenitors, ER-HoxB8, from the bone marrow of C57BL/6 wild-type mice (kindly provided by Andreas Villunger, Medical University of Innsbruck, Austria), was derived by retroviral transduction of HoxB8 and selection in the presence of SCF and β-estradiol as described previously^10^. The estrogen-regulated HoxB8 expression plasmid was a kind gift from Hans Häcker (St. Jude Children's Research Hospital, Memphis, USA, MTA signed on January 23, 2018). The progenitors were cultured in Opti-MEM (Thermo Fisher) supplemented with 10% FCS (Sigma), 250 μM L-glutamine (Corning), 100 U/ml penicillin and 100 μg/ml streptomycin (Corning), 30 mM β-mercaptoethanol (Sigma), 1 μM β-estradiol (Sigma) and 1% supernatant from SCF-producing Chinese hamster ovary (CHO) cells (kindly provided by Hans Häcker, MTA signed on January 23, 2018). After four days of differentiation in the medium without β-estradiol, the cells were harvested and counted as described previously^11^.

### Erythrocyte rosetting assay

P388D1, J774A.1, and neutrophil progenitors were dislodged using a transfer pipet. Enzyme- or chelator-based dissociating agents were not used. The cells (2 × 10^5^) were washed twice in 1 ml of PBS without calcium and magnesium ions (Lonza) and resuspended in 100 µl of PBS. In some experiments, PBS was supplemented with 1 mM EDTA or 2 mM divalent ions (Ca^2+^, Mg^2+^, or Mn^2+^), with the additives present during all steps of the procedure. The cells were incubated with the M18 antibody (200 µg/ml) in a 96-well V-bottom plate for 30 min at RT. In experiments on the N-glycans' impact on IgG3 binding, the concentrations of both native and degM18 were 75 µg/ml. Following the incubation, the plate was centrifuged for 5 min at 200×*g*, and the antibody solution was aspirated. The cells were washed three times with PBS, resuspended in 100 µl of PBS and mixed with 100 µl of 1% (hematocrit) standard human B erythrocytes (Regional Centre of Blood Donation and Blood Treatment in Katowice, Poland). After 30 min of incubation at 4 °C, the cells were transferred onto a glass slide, and rosetting and non-rosetting cells were counted using an inverted light microscope (LeicaDM IL LED Fluo). Three different fields were analyzed per sample, which corresponded to counting more than 130 macrophage-like cells. A cluster of one P388D1 or J774A.1 cell with at least three adjacent erythrocytes was counted as a rosetting cell.

### Flow cytometry

A description of the cell preparation is provided in the section “Erythrocyte rosetting assay”. The cells were incubated with 50 µg/ml M18-488 or degM18-488 for 1 h at 4 °C in a 96-well V-bottom plate. Non-labeled M18 or degM18 were used as autofluorescence controls. After the incubation, the cells were washed three times with cold PBS and resuspended in 400 µl of PBS. The samples were analyzed using a BD FACSCalibur flow cytometer. The results were processed with CellQuestPro software or FlowJo_V10. Fluorescence intensity was measured on the FL-1H channel (10,000 cells were counted). Debris and cell aggregates were excluded from the analysis based on forward (FSC) and side scatter (SSC) plots. Next, the FSC vs. FL-1H plot was used to define the gate for autofluorescence (Supplementary Fig. S1).

### Protease treatment of the cells

P388D1 and J774A.1 cells were treated with trypsin (Lonza) or Accutase (Sigma) for 10 min at 37 °C. The proteases were used at the concentration recommended for cell release. In some experiments, heat-denatured Accutase was used. The cells were washed twice with PBS. IgG3 binding to the treated and control cells was then analyzed using the erythrocyte rosetting assay and flow cytometry.

### Blocking antibodies

IgG3 binding to P388D1 and J774A.1 cells was analyzed in the presence of antibodies that block Fc receptors. FcγRII and FcγRIII were blocked with rat anti-mouse CD16/CD32 Fc-block 2.4G2 (20 µg/ml; BD). FcγRI and FcγRIV were blocked with mouse IgG2a (20, 50 or 100 µg/ml, Sigma, cat. #M5409). The putative non-canonical Fc receptor ITGB1 was blocked with a hamster HMβ1-1 anti-mouse CD29 antibody (20 µg/ml, BD). The cells were incubated with the blocking antibodies for 30 min at RT. The mIgG3 or its derivative was then added directly to the cells with blocking antibodies, followed by the rosetting assay or flow cytometry analysis.

### Statistical analysis

Cochran–Mantel–Haenszel test or Fisher's exact test were used for statistical analysis of the results from rosetting assays. Cochran–Mantel–Haenszel test was used for analysis of results from independent experiments, Fisher's exact test was used for experiments done once. The alpha level for all tests was 0.001. The calculated p value is provided on figures only if it is below the alpha level. Spreadsheets available on http://www.biostathandbook.com/cmh.html and https://www.graphpad.com/quickcalcs/contingency1.cfm were used for the calculations.

## Results

### J774A.1 and P388D1 cells express a receptor for mIgG3

**Figure 1:**
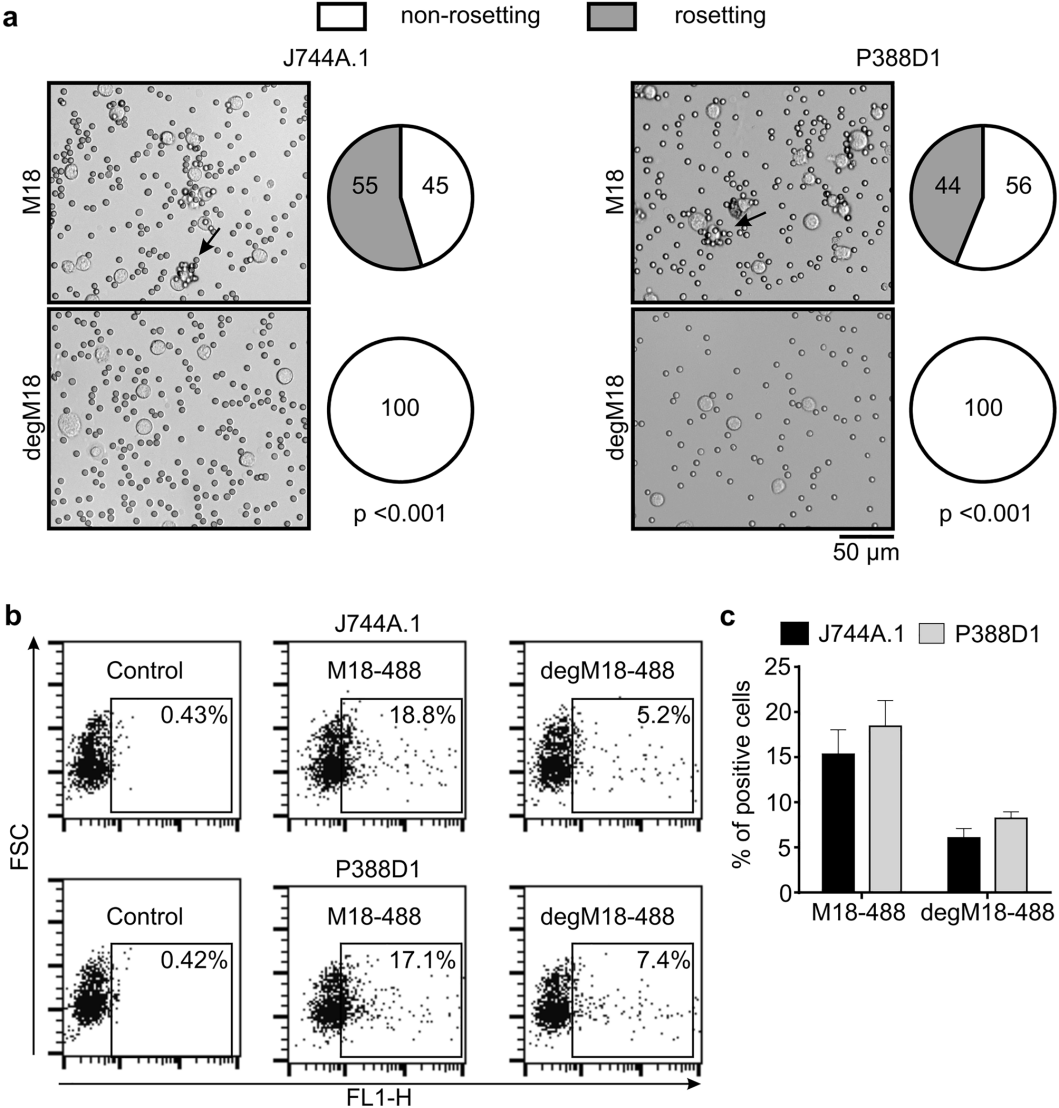
Deglycosylation of mIgG3 prevents its binding to J774A.1 and P388D1 cells. (**a**) Erythrocyte rosetting around J774A.1 or P388D1 cells coated with the M18 mIgG3 antibody specific to antigen B of the human ABO blood group system. The cells were coated with native or deglycosylated M18 (degM18). The small cells are group B human erythrocytes, whereas the large bright cells are J774A.1 or P388D1 cells. The arrows point to exemplary rosettes. Representative microscope images of three independent experiments are shown. The pie charts display the percentage of rosetting and non-rosetting cells observed in three different fields in the same representative experiment. (**b**,**c**) Binding of M18-488 to J774A.1 or P388D1 cells analyzed using flow cytometry. The cells were stained with fluorescently labeled native or degM18. The dot plots show representative results of three independent experiments. (**c**) Average values ± SD of the three independent experiments described in (**b**).

We confirmed the findings by Saylor et al.^6^ that J774A.1 cells express a receptor that is able to bind antigen-free mIgG3 (Fig. 1). We observed erythrocyte rosetting around J774A.1 cells precoated with the erythrocyte-specific mIgG3 M18. Moreover, M18 labeled with DyLight 488 bound to the cells, as demonstrated by flow cytometry. Similar results were obtained with the P388D1 cells (Fig. 1).

We observed heterogeneity in mIgG3 binding. Depending on the experiment, 10–50% of the analyzed J774A.1 or P388D1 cells did not form rosettes, supposedly due to the low expression of the mIgG3 receptor. To eliminate low-binders and select high-binders from the cell populations, we generated clones of single P388D1 cells using the limiting dilution method. Although individual clones differed in the efficacy of rosette formation (Supplementary Fig. S2), each clone still contained non-rosetting cells. The clones also differed in the rate of proliferation (data not shown). P388D1 clone #12 was used in some experiments described below because it had an increased rosetting score and its proliferation rate was similar to the parental cell line. However, rosetting efficacy decreased with the number of passages of the clone to the average level of the parental cell line (data not shown).

Macrophage-like cells are sensitive to proinflammatory molecules that profoundly influence gene expression in the cells. Rosetting assays preceded by stimulation of P388D1 cells with LPS revealed that stimulated and control cells bound mIgG3 similarly (Supplementary Fig. S3).

In addition, we verified whether neutrophil progenitors and differentiated neutrophils^11^ formed rosettes after coating with mIgG3. We did not observe rosettes either around the neutrophils or their progenitors, in contrast to the macrophage-like cells (positive control), which formed rosettes (Supplementary Fig. S4). We also tested if the putative mIgG3 receptors, FcγRI, FcγRII/FcγRIII, and ITGB1, are expressed in the differentiated neutrophils and in the macrophage-like cells. We confirmed the expression of FcγRII/FcγRIII and ITGB1 in the neutrophils and in the macrophage-like cells, whereas FcγRI was detected only in the macrophage-like cells (Supplementary Figs. S5, S6 and S7).

Taken together, we confirmed that antigen-free mIgG3 binds to the surface of macrophage-like J774A.1 and P388D1 cells. Based on the obtained results, the receptor complex that binds antigen-free mIgG3 is present on monocytes/macrophages. In the case of neutrophils we did not observe rosetting; therefore the receptor complex is absent on neutrophils or its expression is too low to allow rosetting.

### Deglycosylation of mIgG3 affects its binding to J774A.1 and P388D1 cells

IgG binding to known FcγRs depends on N-glycans^12^. Thus, we verified whether mIgG3 binding to J774A.1 and P388D1 cells was also dependent on N-glycans attached to the CH2 domain of the antibody. Deglycosylated mIgG3 M18 (degM18) did not induce erythrocyte rosetting around J774A.1 or P388D1 cells (Fig. 1). Previously we reported that deglycosylation does not influence M18 binding to erythrocytes^13^. Therefore, the lack of rosette formation in the presence of degM18 excluded the interaction between degM18 and the surface of the cells.

We confirmed the effect of mIgG3 deglycosylation on its binding to J774A.1 and P388D1 cells in a flow cytometric analysis. Consistent with the results of the rosetting assay, deglycosylation of M18 sharply diminished its binding to J774A.1 and P388D1 cells. A substantially lower percentage of the cells was stained with degM18-488 than with native M18-488 (Fig. 1).

### Protein A does not influence mIgG3 binding to macrophage-like cells

The known FcγRs do not compete with protein A to bind to IgG^14^. We verified whether the binding of mIgG3 to macrophage-like cells can be prevented by protein A. Using flow cytometry we demonstrated that protein A did not influence the binding of M18-488 to J774A.1 or P388D1 cells (Supplementary Fig. S8). Thus, it is likely that receptors on the macrophage-like cells and protein A bind different residues in mIgG3 constant region.

### Susceptibility of the IgG3 receptor to proteolysis

**Figure 2:**
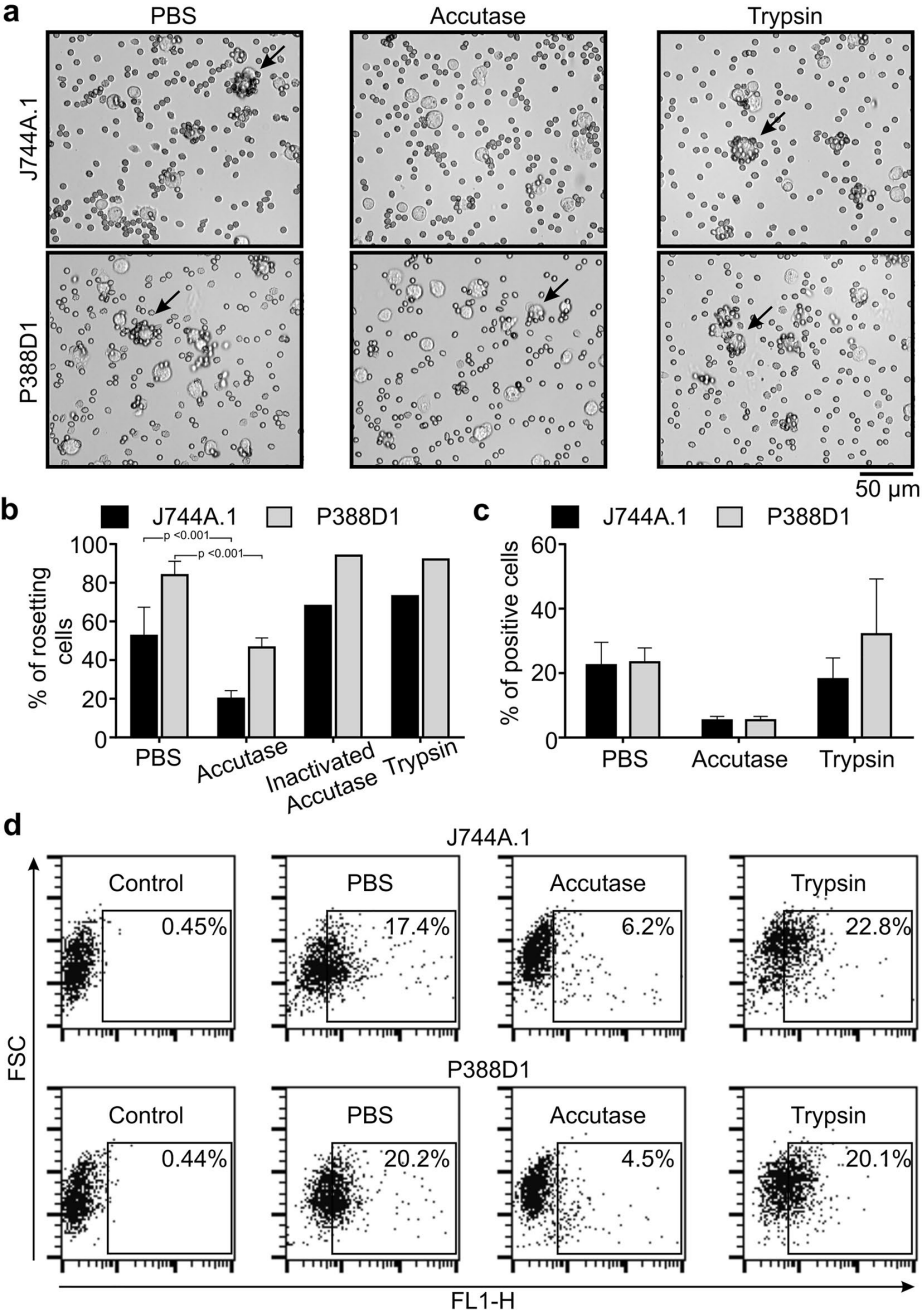
Binding of mIgG3 to J774A.1 and P388D1 is diminished after Accutase treatment of the cells. (**a**,**b**) Rosetting of erythrocytes around J774A.1 and P388D1 cells was induced by mIgG3 M18 specific to antigen B of the human ABO blood group system. Control and protease-treated cells were coated with M18 mIgG3 and then incubated with group B erythrocytes. (**a**) Representative microscope images of three independent experiments are shown. The arrows point to exemplary rosettes. (**b**) The chart presents average values ± SD from two independent rosetting experiments performed for the negative control (PBS) and Accutase treatment and from one experiment performed for inactivated Accutase and trypsin. Three randomly chosen fields were analyzed for each sample in each experiment. (**c**,**d**) Flow cytometry analysis of M18-488 binding to J774A.1 or P388D1 cells. The cells were treated with the enzymes and then stained with fluorescently labeled M18-488. (**c**) Average values ± SD from two independent experiments are shown. (**d**) Representative dot plots from the flow cytometry analysis. (**a**–**d**) The clone #12 of P388D1 was used in these experiments.

Accutase is often used as an alternative to trypsin for detaching adherent cells from a culture dish. We analyzed mIgG3 binding to the macrophage-like cells treated with trypsin or Accutase. The analyses showed that pretreatment of J774A.1 and P388D1 with Accutase, but not trypsin, prevented the binding of mIgG3 to the cells (Fig. 2).

At least one of the known FcγRs is shed from the cell surface by the disintegrin and metalloproteinase domain-containing protein 17 (ADAM17)^15^. We analyzed mIgG3 binding to ADAM17^−/−^-P388D1 cells generated by CRISPR-Cas9 technology. We did not observe any difference between ADAM17^−/−^ cells and the parental cell line in the rosetting assay (Supplementary Fig. S9). The results indicate that the ADAM17 knockout does not influence the binding of mIgG3 to the surface of the cells.

### Involvement of putative receptors in the mIgG3 binding

**Figure 3:**
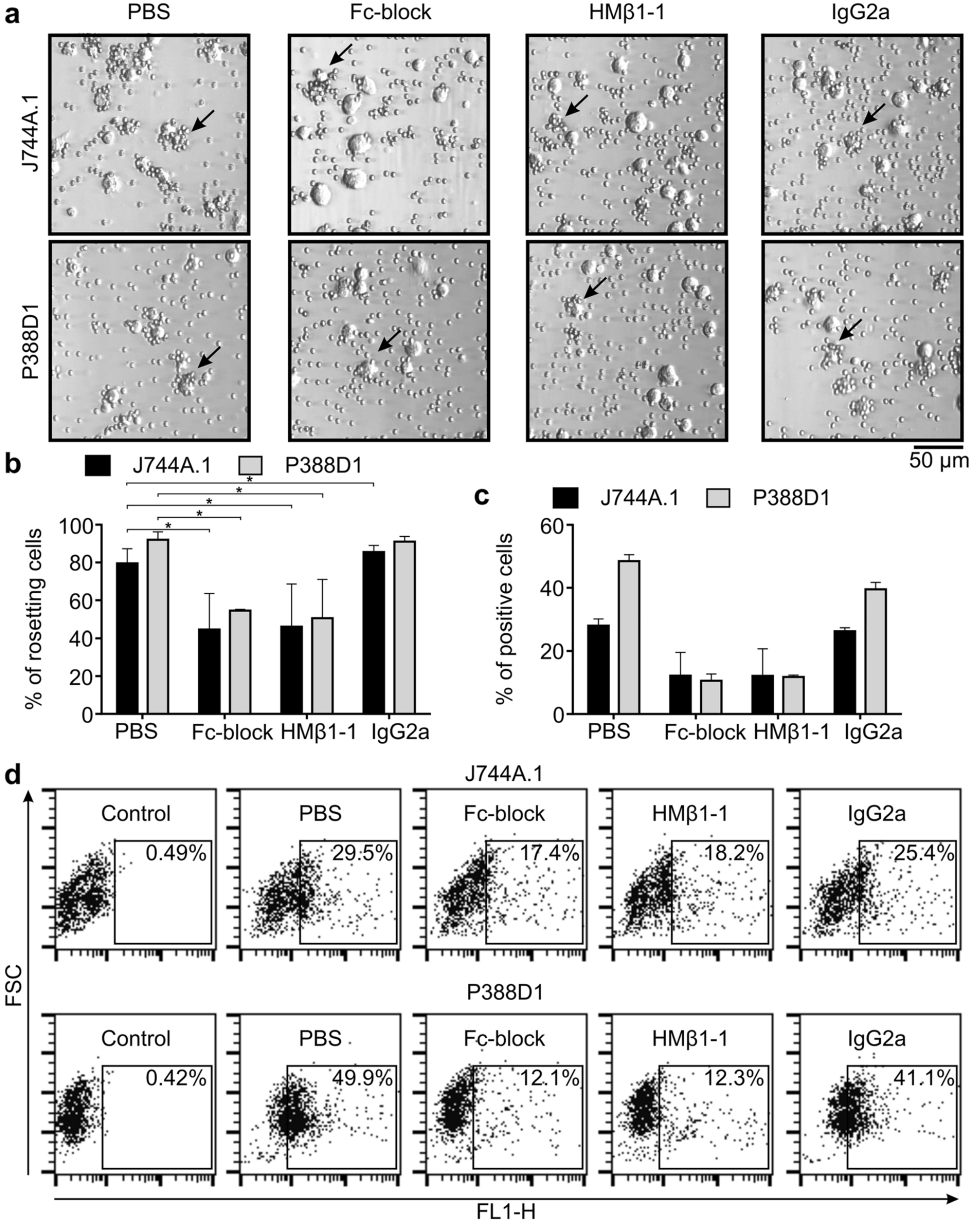
Binding of mIgG3 to J774A.1 or P388D1 cells in the presence of antibodies blocking the putative receptors of mIgG3. Fc-block inhibits binding to FcγRII and FcγRIII, HMβ1-1 blocks ITGB1, and IgG2a is a competitor for IgG binding to FcγRI and FcγRIV. (**a**,**b**) Rosetting of erythrocytes around J774A.1 and P388D1 cells coated with mIgG3 M18, which binds antigen B of the ABO blood group system. The cells were pre-incubated with the blocking antibodies, then coated with M18 mIgG3 and analyzed in the rosetting assay. (**a**) Representative microscope images of two independent rosetting experiments are shown. Exemplary rosettes are indicated with arrows. (**b**) Average values ± SD from two independent rosetting experiments are shown. Three randomly chosen fields were analyzed for each sample in each experiment, *p < 0.001. (**c**,**d**) Flow cytometry analysis of M18-488 binding to J774A.1 or P388D1 cells. The cells were pre-incubated with the blocking antibodies and then stained with M18-488 in the presence of the blocking antibodies. (**c**) Average values ± SD from two independent experiments are shown. The same chart extended with results for pairs of the blockers is presented in Supplementary Fig. S14. (**d**) Representative dot plots from the cytometry analysis. (**a**–**d**) The clone #12 of P388D1 was used in these experiments.

Hawk et al. suggested the existence of different mIgG3 receptors^5^. We analyzed mIgG3 binding in the presence of antibodies blocking the putative mIgG3 receptors. Rosetting and M18-488 binding were substantially diminished in the presence of antibodies blocking FcγRII/FcγRIII or ITGB1 (Fig. 3). A competitive blockade of FcγRI and FcγRIV with mouse IgG2a, a high-affinity ligand of these receptors, reduced M18-488 binding only when IgG2a was used at a concentration equal or higher than that of mIgG3 (Fig. 3 and Supplementary Fig. S10). These results indicate that FcγRII, FcγRIII and ITGB1 are involved in the binding of free mIgG3. IgG2a binds to FcγRII and FcγRIII with micromolar affinity; thus, the effect of a high concentration of IgG2a on mIgG3 binding might be a result of the competitive blockade of FcγRII and FcγRIII.

We showed that the treatment of the cells with Accutase affects mIgG3 binding. We also confirmed that FcγRII, FcγRIII and ITGB1 are involved in mIgG3 binding. We tried to link these two observations by verifying whether Accutase treatment reduced the expression of FcγRII/FcγRIII or ITGB1 on the cell surface.

The expression of the receptors on control and Accutase-treated J774A.1 or P388D1 cells was compared in western blotting. The membranes were probed with polyclonal antibodies raised against extracellular domains of the receptors. A polyclonal anti-FcγRII/FcγRIII antibody revealed that Accutase cleaved FcγRII/FcγRIII on P388D1, whereas trypsin did not affect these receptors (Supplementary Fig. S7). FcγRI was identically cleaved by both enzymes. Therefore, the results support the involvement of FcγRII/FcγRIII in mIgG3 binding.

In the same assay, a polyclonal anti-ITGB1 antibody detected two variants of ITGB1 with molecular weights above and below 130 kDa. The < 130 kDa variant of ITGB1 was affected neither by Accutase nor trypsin but the > 130 kDa variant was cleaved by both enzymes (Supplementary Fig. S7). Since both enzymes cut ITGB1, we rejected the hypothesis that ITGB1 is an Accutase-sensitive receptor for mIgG3. To explore further the involvement of ITGB1 in mIgG3 binding, we tried to explain why the ITGB1-blocking antibody (HMβ1-1) prevents binding of mIgG3 to the macrophage-like cells.

It has been demonstrated that some anti-integrin antibodies induce internalization of their targets^16,17^. To verify if HMβ1-1 induces ITGB1 internalization, we incubated the P388D1 cells with HMβ1-1 or with a matched isotype control, and then we stained the cells with the polyclonal anti-ITGB1 antibody. Flow cytometry analysis revealed that ITGB1 expression on the HMβ1-1-treated cells was lower than on the control cells (Supplementary Fig. S11). The results indicate that HMβ1-1 induced internalization of the target. Since ITGB1 is considered as a part of a mIgG3-binding complex^5^, other proteins involved in mIgG3 binding may be co-internalized with ITGB1 upon incubation with the HMβ1-1 antibody.

### mIgG3 binding to cell surface in the presence of divalent ions

Calcium-dependent binding of mIgG3 to macrophage-like cells would support the hypothesis that ITGB1 is a part of a complex that binds mIgG3. Indeed, Hawk et al. demonstrated a considerable increase in mIgG3 binding in the presence of Ca^2+^^5^. We analyzed rosetting and M18-488 binding to J774A.1 and P388D1 cells in the presence of divalent ions. Contrary to the report by Hawk et al., calcium ions influenced neither rosetting nor M18-488 binding to the cells (Supplementary Fig. S12). Similar results were obtained with Mg^2+^ (Supplementary Fig. S13), whereas 1 mM Mn^2+^ was toxic for the cells and it was therefore excluded from the analysis (data not shown).

## Discussion

Mouse IgG3s are rarely generated using hybridoma technology and are not easy targets for investigators. The mIgG3 molecules oligomerize via their Fc regions, and the ability of oligomerization results in a high affinity for polyvalent antigens^18^. Moreover, many of mIgG3s are cryoglobulins—they reversibly precipitate at low temperatures^19^. This sporadic occurrence is the main reason for the low interest in mIgG3 among scientists. It has been shown that the reactivity of common immunoglobulin detection reagents with mIgG3 is low^20^. Thus, the importance of mIgG3 in mouse immunity might be underestimated because of inefficient detection methods.

Many authors consider mIgG3 as a subtype with effector functions restricted to the complement cascade. This opinion is supported by the lack of interaction between the known mouse FcγRs and mIgG3 in biophysical assays^8,21^. However, in vitro and in vivo effects triggered by mIgG3 suggested the existence of a mIgG3-specific receptor^5,6,22^. Despite many efforts made mainly by Casadevall's group, this receptor has not been identified.

In 2019, Hawk et al. published a paper showing that ITGB1 is involved in the phagocytosis of *Cryptococcus neoformans* opsonized with mIgG3^5^. The authors demonstrated that knockout or blockade of ITGB1 interferes with mIgG3 binding to mouse macrophages. They suggested that ITGB1 is a part of mIgG3-binding complex, because CHO cells co-expressing mouse ITGB1 and ITGA4 bound mIgG3, whereas CHO cells expressing only ITGB1 interacted with mIgG3 comparably as control cells.

We confirmed that blockade of ITGB1 reduces mIgG3 binding to the J774A.1 and P388D1 cells. In contrast to Hawk et al., we did not observe any change in mIgG3 binding to the cells in the presence of divalent ions. For the majority of integrin complexes, Mn^2+^ and Mg^2+^ activate integrins and promote binding to their substrates, whereas Ca^2+^ usually keeps integrins in an inactive state^23,24^. We demonstrated that neither Ca^2+^ nor Mg^2+^ influenced mIgG3 binding to the cells. Thus, the results suggest that mIgG3 binding does not depend on the state of ITGB1. ITGB1 forms or is at least required in the assembly of a mIgG3-specific complex. According to our findings, in addition to ITGB1, FcγRII and FcγRIII are also possibly involved in the binding of antigen-free mIgG3.

Gavin et al. identified FcγRI as a macrophage receptor for mIgG3, but only for antibodies bound to a polyvalent antigen, e.g., erythrocyte coated with mIgG3 antibodies^7^. Pre-incubation of the macrophages with monomeric IgG2a prevented the binding of the mIgG3-polyvalent antigen complex to the cells. In that same work, fluorescently labeled mIgG3 did not bind to FcγRI-expressing CHO cells. In our experiments, we used a different methodology: we coated macrophage-like cells with mIgG3, washed out unbound immunoglobulins, and then assessed the bound mIgG3 by flow cytometry or a rosetting assay. Therefore, we demonstrated that a receptor binding antigen-free mIgG3 is present on the surface of J774A.1 and P388D1 cells. Contrary to Gavin et al., in our experiments, the blockade of FcγRI with mouse IgG2a did not prevent mIgG3 binding to the cells. Moreover, we demonstrated that FcγRI is cleaved by both Accutase and trypsin, whereas only treatment with Accutase affected binding of mIgG3 to the macrophage-like cells; therefore, we disproved the involvement of FcγRI in binding of antigen-free mIgG3.

We focused exclusively on the binding of mIgG3 to the surface of the macrophage-like cells, whereas Hawk et al. employed a *C. neoformans* phagocytosis model for similar analyses^5^. Phagocytosis of a pathogen opsonized with an IgG is a multi-step phenomenon that depends on the cooperation of phagocytic receptors and integrins. Recognition and initial binding of the opsonized pathogen is mediated by phagocytic receptors, e.g., FcγRs that in turn activate integrins. The activated integrins bridge the phagocytic receptors and generate a diffusion barrier for phosphatases which extinguish signal transduction^25^. Therefore, phagocytic receptors initiate phagocytosis, but the integrins orchestrate phagocytic cup formation and engulfment. Integrin blockade or knockout might disturb signaling from mIgG3-specific phagocytic receptors and consequently affect phagocytosis efficacy, as well as the tight binding of an opsonized particle.

A similar scenario is likely in the erythrocyte rosetting model. Initial binding of erythrocytes to macrophage-like cells with FcRs loaded with erythrocyte-specific mIgG3 might be followed by tight, ITGB1-dependent binding of the erythrocytes. Integrins could enhance binding to an erythrocyte by bridging the FcRs.

As discussed, the impairment of phagocytosis and erythrocyte rosetting observed upon ITGB1 blockade could be explained based on the available knowledge. However, the ITGB1 blockade resulted also in the decreased binding of free mIgG3 to the surface of macrophage-like cells. This phenomenon was discovered by Hawk et al.^5^ and confirmed by us. For ITGB1 inhibition, we used the HMβ1-1 antibody that blocks the binding of the integrin complex to collagen, laminin, and fibronectin. The HMβ1-1 antibody also inhibits proliferation of splenocytes activated by an anti-CD3 antibody^26^. The precise mechanism of action of the HMβ1-1 antibody is unknown, but we demonstrated that HMβ1-1 reduces surface expression of ITGB1 presumably by inducing internalization of the target. Internalization of ITGB1 upon binding to the antibody seems acceptable because it was described previously for other integrin complexes^16,17^. We propose that an unknown mIgG3 receptor is internalized together with the ITGB1-HMβ1-1 complex. Consequently, a reduction in the surface expression of the mIgG3-specific receptor might decrease the binding of mIgG3 to macrophage-like cells.

Our results also indicate that the well-known Fc receptors FcγRII and FcγRIII are involved in mIgG3 binding. In the original research, in which interactions between mIgG3 and the Fc receptors were analyzed, mIgG3 did not bind any of the receptors^8^. However, we demonstrated that blockade of FcγRII and FcγRIII affects mIgG3 binding to the macrophage-like cells. The experiments testing protease susceptibility further supported the involvement of FcγRII and FcγRIII in the binding of mIgG3. Moreover, we showed that mIgG3 binding depends on N-glycans present on the CH2 domain of the antibody. N-glycans are essential in the interaction between other IgG subclasses and the FcγRs. Thus, the lack of binding of deglycosylated mIgG3 to macrophage-like cells supports the involvement of FcγRII and FcγRIII in mIgG3 recognition.

Among the known integrins, only α_M_β_2_ comprises a lectin-like domain specific to beta-glucan^27^. There are also reports demonstrating that the lectin-like domain recognizes N-glycans^28,29^. The binding site for the saccharides is in the membrane proximal part of the α_M_ chain and it has not been precisely defined^30^. The α_M_ chain does not dimerize with ITGB1; thus, it is unlikely that the lectin-like domain of α_M_ binds N-glycan of mIgG3. However, there is a substantial similarity in the membrane proximal fragment within all α chains. Therefore, the α chains interacting with ITGB1 may comprise undiscovered lectin-like binding sites resembling the one identified in α_M_ chain.

Hawk et al. suggested heterogeneity in mIgG3 preparations, implicating that different fractions of mIgG3 can be captured by different receptors^5^. We believe that the mIgG heterogeneity results from differences in the N-glycosylation. It is possible that the binding of mIgG3 to FcRs is mainly influenced by a structure of N-glycans on the antibody.

The blockade of the FcγRII and FcγRIII receptors with Fc-block reduced mIgG3 binding to a value observed when ITGB1 was blocked. This result indicates that FcγRII/FcγRIII and ITGB1 are not redundant mIgG3 receptors. Neither Fc-block nor the HMβ1-1 antibody completely abolished mIgG3 binding. The macrophage-like cells bound mIgG3 even upon dual blockade with Fc-block and HMβ1-1, suggesting that mIgG3 interacts also with another receptor on the surface of the cells.

In addition, we demonstrated for the first time that mIgG3 binding to macrophage-like cells depends on the cell preparation. Cells treated with Accutase did not bind free mIgG3, whereas cell treatment with trypsin did not affect mIgG3 binding. Among the known putative mIgG3 receptors, only FcγRII or FcγRIII were specifically cleaved by Accutase. The unknown mIgG3 receptor is likely a substrate for Accutase, which might be cleaved by the enzyme or even released from the cell surface. This observation is crucial for the appropriate preparation of the cells for experiments exploring the mIgG3 interaction with the cell surface.

## Conclusions

In summary, mouse IgG3 is a rare antibody subtype with a high efficacy in preventing some infectious diseases. An understanding of the immune cell response to pathogens coated with mIgG3 is hampered by a lack of knowledge of the receptors specific to mIgG3. In our work, we confirmed that an unknown mIgG3-specific receptor is expressed on the surface of J774A.1 and P388D1 cells. We demonstrated that blockade of FcγRII/FcγRIII or ITGB1 reduces the binding of mIgG3 to cells. For the first time, we showed that the preparation of cells, as well as antibody glycosylation, should be taken into account in experiments exploring the interaction between mIgG3 and the surface of immune cells. According to our findings, the unknown mIgG3-specific receptor is a substrate for Accutase, which is commonly used for detaching cells from culture vessels.

## Supplementary Information

Supplementary Information.

## Data Availability

All data generated or analyzed during this study are included in this published article (and its Supplementary Information file).
