# Supplementary material for: Mouse IgG3 binding to macrophage-like cells is prevented by deglycosylation of the antibody or by Accutase treatment of the cells
Source: Sci Rep. 2021 May 13;11:10295. doi: 10.1038/s41598-021-89705-3 (PMC8119965; doi:10.1038/s41598-021-89705-3)
Supplement: Supplementary file 1 — Supplementary Information. [file 41598_2021_89705_MOESM1_ESM.pdf]

Mouse IgG3 binding to macrophage-like cells is prevented by deglycosylation of the antibody or by Accutase treatment of the cells

Supplementary Figures

Alicja Karabasz[1], Monika Bzowska[1], Joanna Bereta[1], Maria Czarnek[1], Maja Sochalska[2], and Tomasz Klaus*[1a]

[1]Department of Cell Biochemistry, Faculty of Biochemistry, Biophysics and Biotechnology, Jagiellonian University in Kraków, 7 Gronostajowa, 30-387 Kraków, Poland

[2]Department of Microbiology, Faculty of Biochemistry, Biophysics and Biotechnology, Jagiellonian University in Kraków, 7 Gronostajowa, 30-387 Kraków, Poland

*corresponding author, e-mail: tomasz.klaus@alumni.uj.edu.pl

[a]present address: Pure Biologics Inc., 11 Duńska, 54-427 Wrocław, Poland; e-mail: t.klaus@purebiologics.com

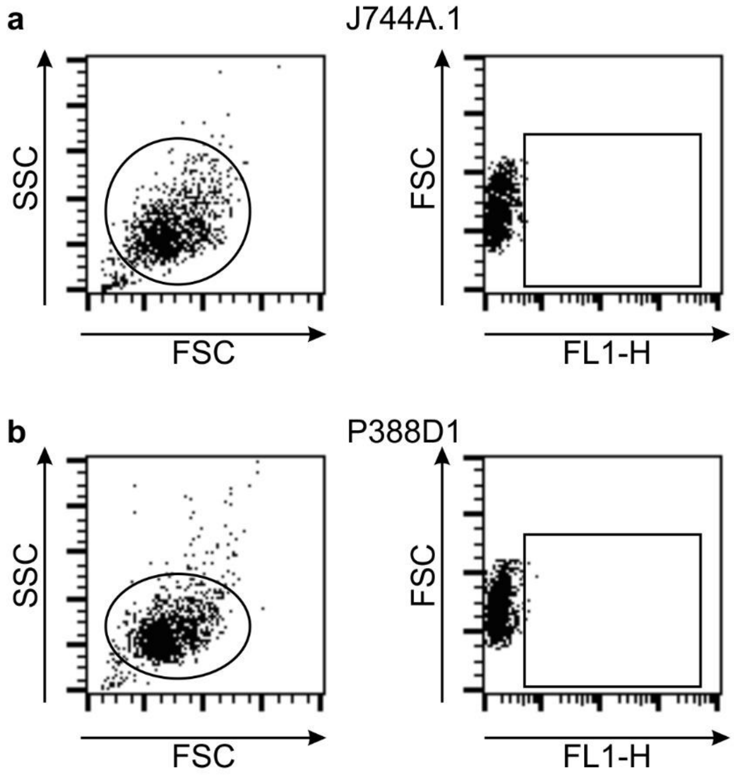

Figure S1 – Gating strategy for **a** – J744A.1 and **b** – P388D1 cells.

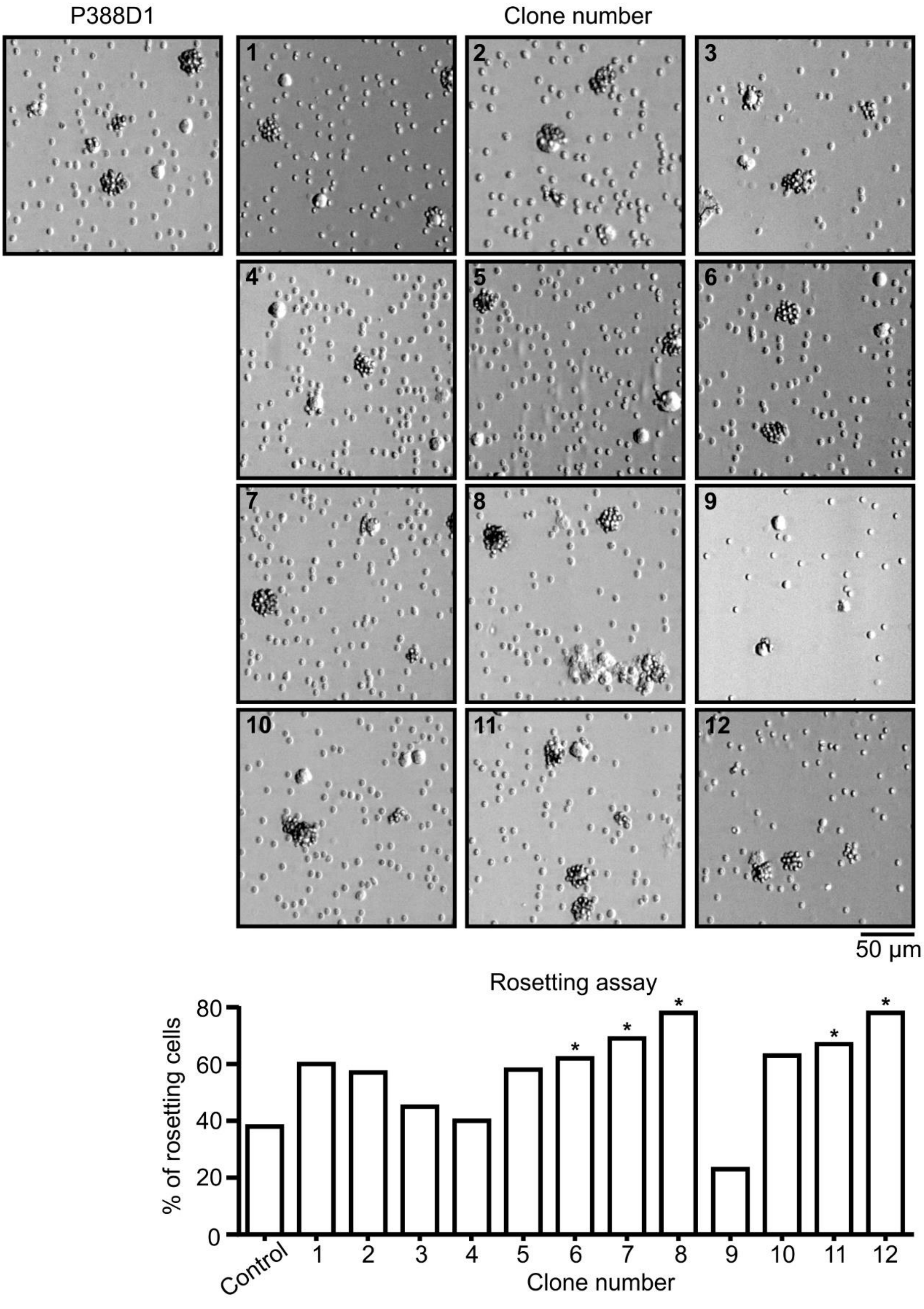

Figure S2 – Binding of mIgG3 to clones derived from single P388D1 cells analyzed in erythrocyte rosetting assay. The cells were coated with M18 mIgG3, followed by incubation with human group B erythrocytes. The experiment was done once. In the statistical analysis the samples were compared to the control, *p < 0.001.

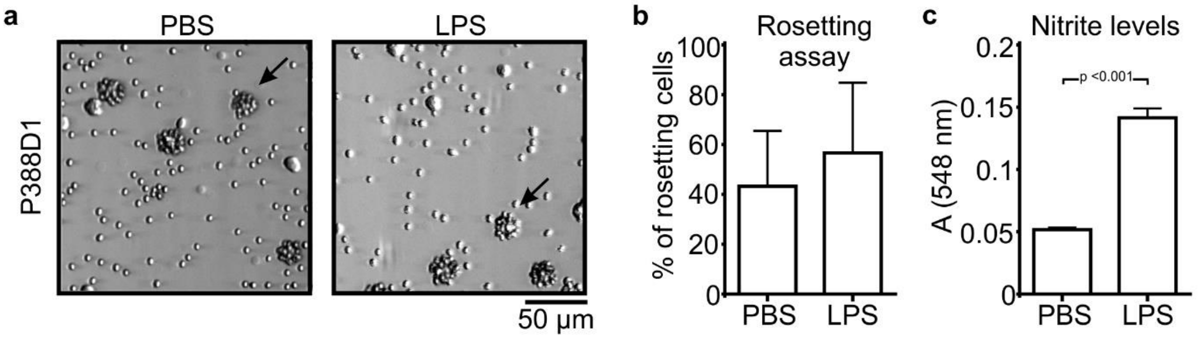

Figure S3 – Binding of mIgG3 to P388D1 cells stimulated with lipopolysaccharide (LPS). **a** and **b** – Erythrocyte rosetting. Before the rosetting assay, P388D1 cells were subjected to overnight serum starvation followed by stimulation with LPS (100 ng/ml, 24 h). PBS was added instead of LPS to the control cells. **a** – Representative microscope images of three independent rosetting experiments are shown. Exemplary rosettes are indicated with arrows. **b** – Average values ± SD from the three independent rosetting experiments. **c** – Increased nitrite levels measured by Griess reaction confirmed activation of P388D1. Average values ± SD from the three independent experiments are shown. The p value was calculated using one-tailed t-test in GraphPad Prism 6.

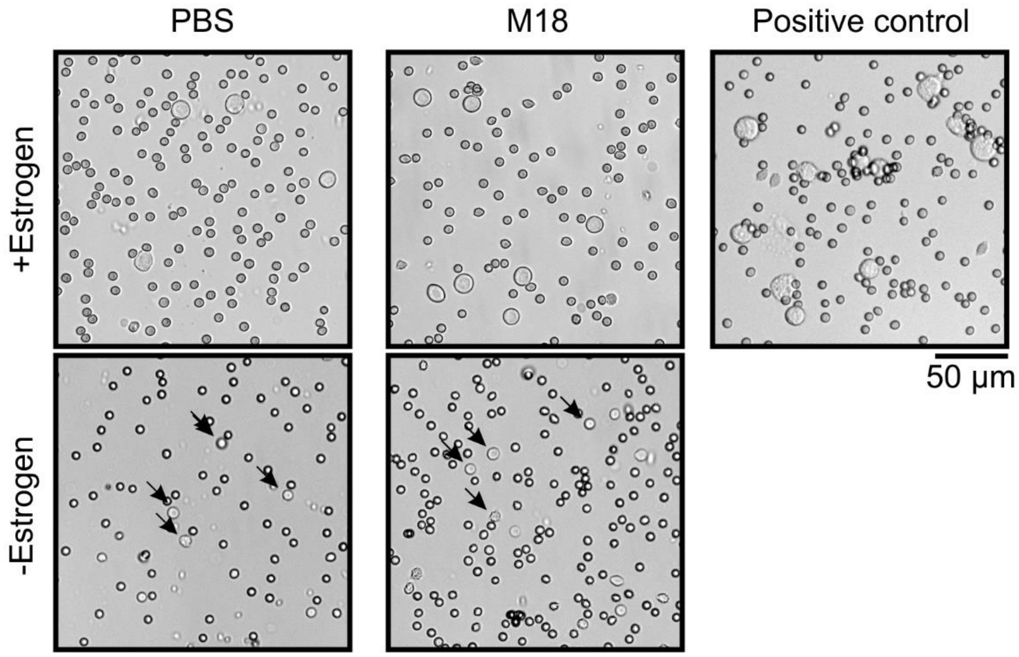

Figure S4 – Mouse IgG3 binding to estrogen-regulated mouse neutrophil progenitors ER-Hoxb8. Neutrophil progenitors were grown in 6-well plates with or without 1 μM β-estradiol (estrogen) for 4 days according to the protocol [1]. Rosetting assay was performed using non-differentiated (control) and differentiated cells. Exemplary neutrophils are indicated with arrows. Positive control for the rosetting assay was done using P388D1 cells. Representative results of three independent experiments are shown.

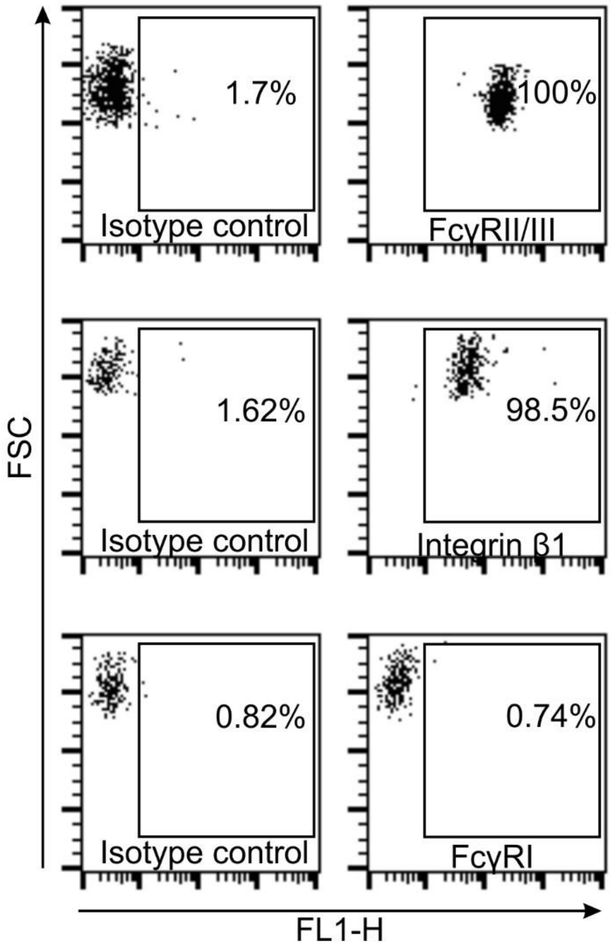

Figure S5 – FcγRI, FcγRII/III, and ITGB1 expression on differentiated neutrophils. The neutrophils were stained with rat anti-FcγRII/III (Fc-block, 2 μg/ml, BD Pharmingen), hamster anti-ITGB1 (HMβ1-1, 2 μg/ml, BD), or goat anti-FcγRI (AF2074, 2 μg/ml, R&D Systems). Matched isotype controls were used to demonstrate non-specific binding of the primary antibodies. The bound antibodies were detected with secondary antibodies conjugated with Alexa Fluor 488 (goat anti-rat IgG, goat anti-hamster IgG, or donkey anti-goat IgG, all from Thermo Fisher, 10 μg/ml). Non-viable cells were excluded from the analysis using staining with 7-Amino-Actinomycin D (7-AAD, 2.5 μg/ml). Representative results of two independent experiments are shown.

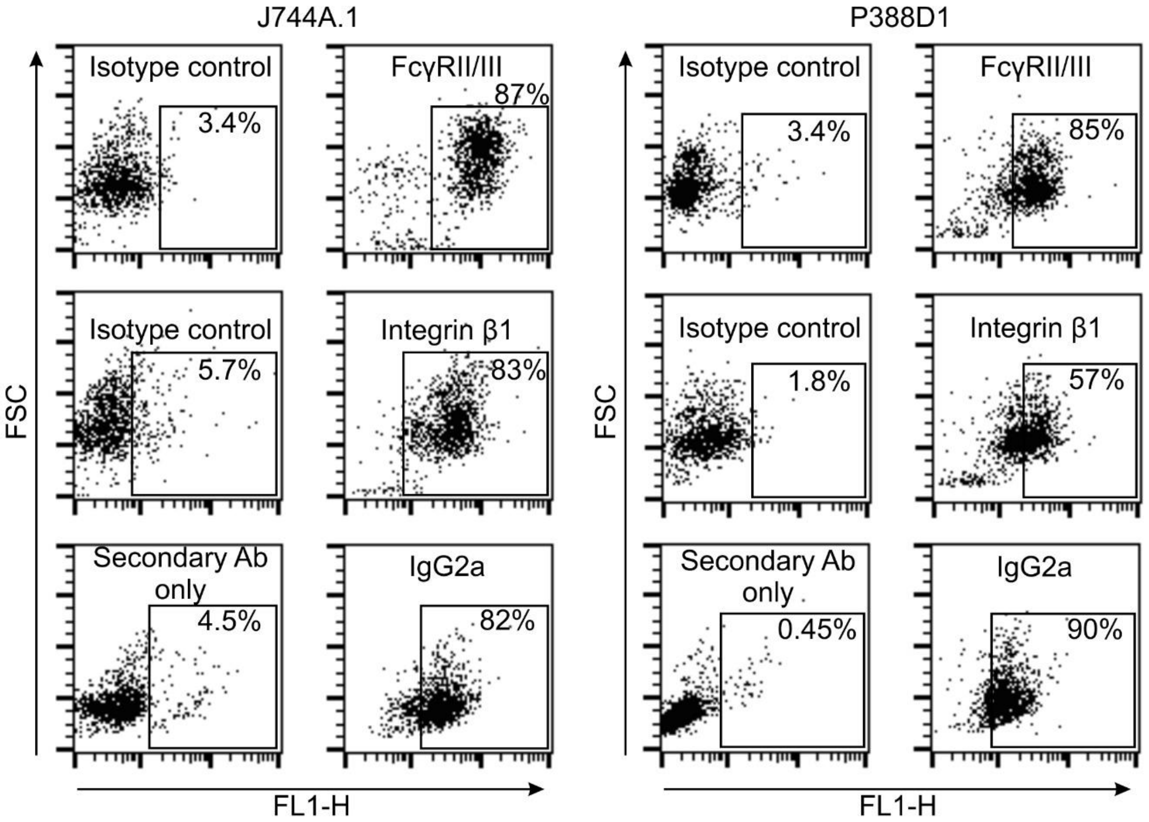

Figure S6 – FcγRI, FcγRII/III, and ITGB1 expression on J744A.1 and P388D1 cells – flow cytometry analysis. The macrophage-like cells were stained with rat anti-FcγRII/III (Fc-block, 2 μg/ml, BD Pharmingen), hamster anti-ITGB1 (HMβ1-1, 2 μg/ml, BD). FcγRI was detected using its ligand – mouse IgG2a (2 μg/ml, Sigma, cat. #M5409). Matched isotype controls were used to demonstrate non-specific binding of the rat and hamster primary antibodies. The bound antibodies were detected with secondary antibodies conjugated with Alexa Fluor 488 (10 μg/ml, goat anti-rat IgG, Thermo Fisher; 10 μg/ml, goat anti-hamster IgG, Thermo Fisher; or 1:100 donkey anti-mouse IgG, Jackson Immunoresearch). Non-viable cells were excluded from the analysis using staining with 7-Amino-Actinomycin D (7-AAD, 2.5 μg/ml). The clone #12 of P388D1 was used in the experiments.

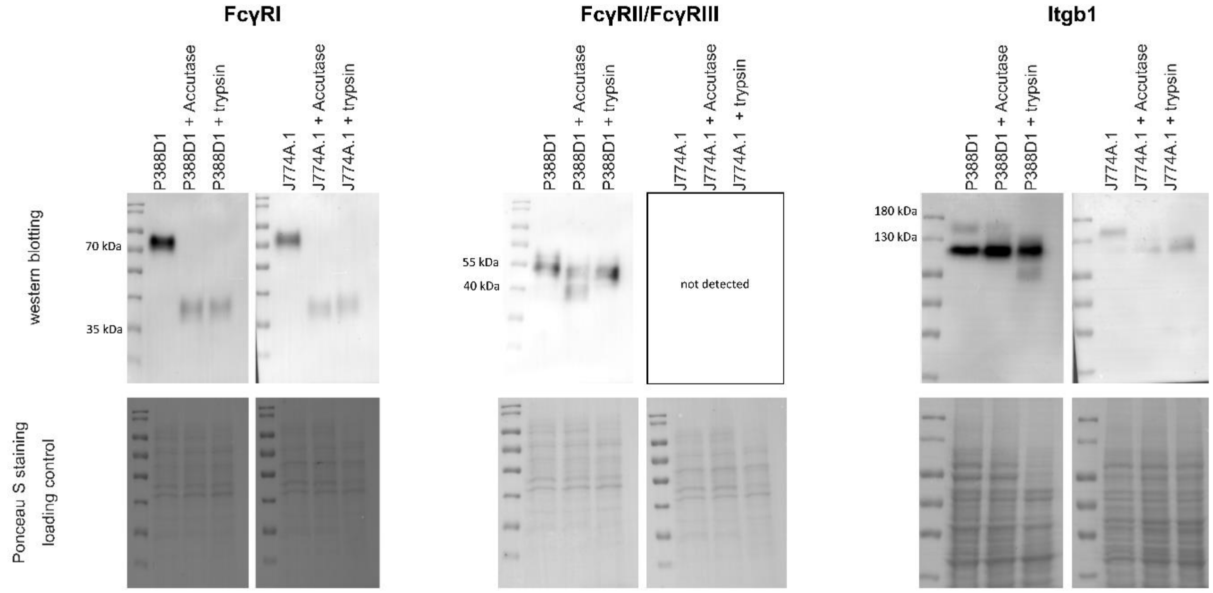

Figure S7 – FcγRI, FcγRII/III, and ITGB1 expression on J774A.1 and P388D1 cells – western blotting analysis. The control and enzyme-treated cells were lysed in RIPA buffer. 20-40 μg of lysate total protein was resolved in SDS-PAGE, then transferred onto PVDF membrane and probed with polyclonal antibodies to FcγRI (AF2074, R&D Systems, 0.5 μg/mL), FcγRII/III (AF1460, R&D Systems, 0.5 μg/mL) or ITGB1 (AF2405, R&D Systems, 0.5 μg/mL). The primary antibodies were detected with rabbit anti-goat IgG antibody conjugated with HRP (A4174, Sigma, 1:10,000). Representative results of at least two independent experiments are shown. Figure S15 presents uncropped images of the blots.

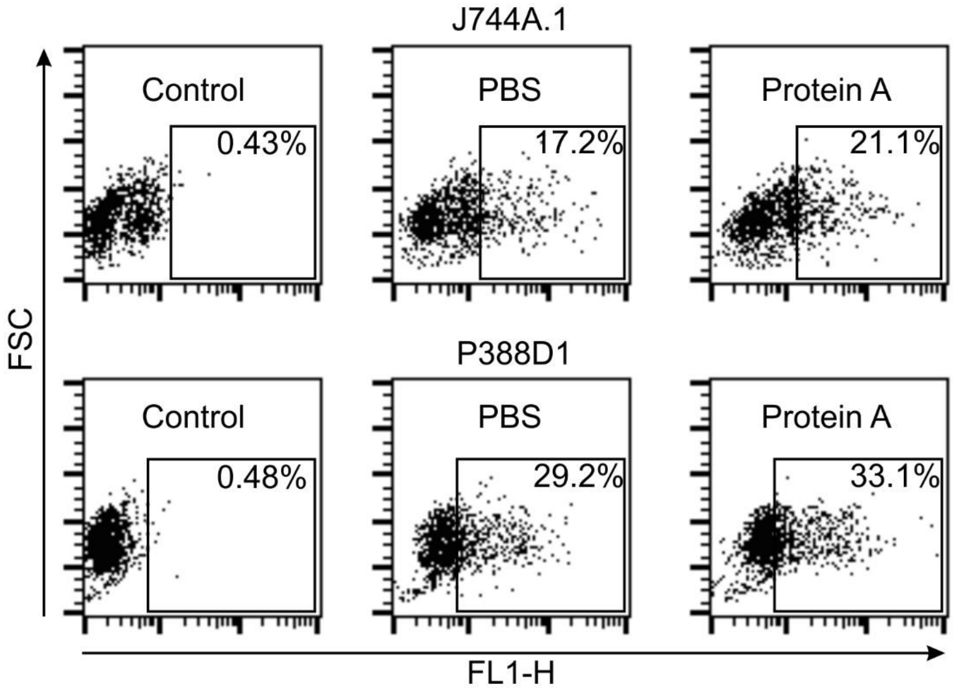

Figure S8 – Binding of mIgG3 to J774A.1 and P388D1 cells in the presence of protein A. The cells were stained with mIgG3 M18-488 in the presence of protein A (67 μg/ml) or in control solution (PBS) and analyzed using flow cytometry. The experiment was done once.

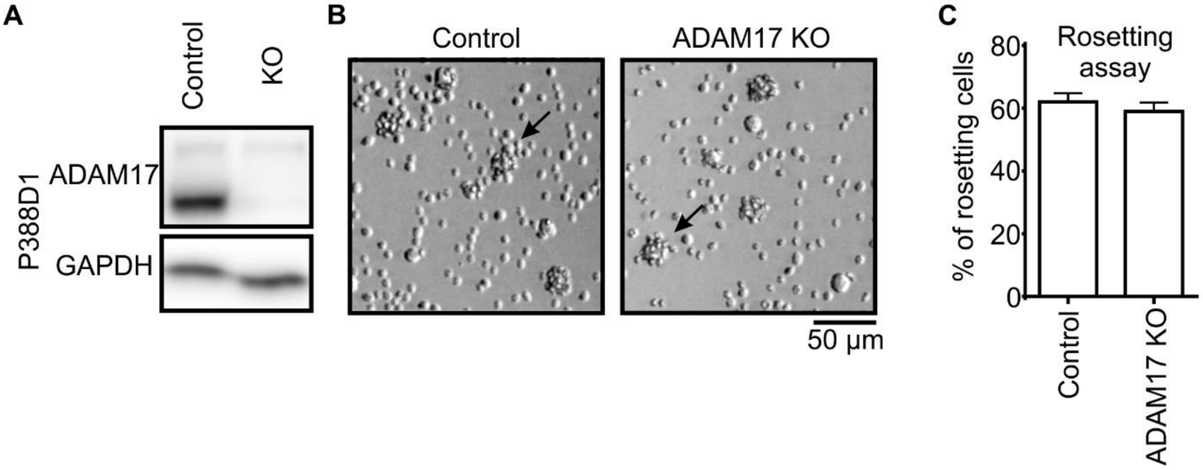

Figure S9 – The knock-out of ADAM17 does not affect mIgG3 binding to P388D1 cells. **A** – ADAM17 was efficiently knocked-out in P388D1 cells using CRISPR-Cas9 technology. SpCas9 was amplified from pX330-U6-Chimeric_BB-CBh-hSpCas9 (a gift from Feng Zhang, Addgene plasmid #42230; http://n2t.net/addgene:42230; RRID:Addgene_42230 [2] and cloned into pJET1.2 (Thermo Scientific). After linearization with XbaI, Cas9 mRNA was in vitro transcribed using the HiScribe™ T7 ARCA mRNA Kit (with tailing) (NEB). sgRNA were designed to target the following sequences (PAM in bold): Adam17 exon 1: GTGGTGGACGGGAACATGAGG**CGG** (sense strand); Adam17 exon 2: GTGCTGCTGAATATTAGCTA**AGG** (antisense strand), Adam17 exon 3: GAAAGCGAGTACAGCGTGAAG**TGG** (sense strand), GFP: GCTGGAGTTCGTGACCGCCGC**CGG** (sense strand). Oligonucleotides corresponding to the Adam17- or EGFP-targeting portion of sgRNAs were cloned into pX330- U6-Chimeric_BB-CBh-hSpCas9. sgRNAs were PCR-amplified, and purified PCR products were *in vitro* transcribed using T7 polymerase (NEB). Cas9 mRNA and sgRNAs were purified by LiCl precipitation. P388D1 cells were electroporated with 2 µg of Cas9 mRNA using Gene Pulser II (Bio-Rad) and 0.4 cm gap cuvettes (Bio-Rad) (electroporation parameters: 300 V, 300 µF, infinite resistance) in electroporation buffer (100 mM sodium phosphate, 10 mM MgCl 2 , 5 mM KCl, 20 mM HEPES, 50 mM sodium succinate; pH 7.2). After 4 h, cells were electroporated with 1 µg of sgRNA targeting Adam17 or EGFP (control cells). Cells were allowed to regenerate in culture (5-7 days), and the electroporation was repeated with another sgRNA (three in total). Figure S15 presents uncropped images of the blots. **B** – Rosetting assay. Wild type and ADAM17 knock-out P388D1 cells were coated with mIgG3 specific to antigen B of the human ABO blood group system. Then, the cells were incubated with B group erythrocytes. Exemplary rosettes are indicated with arrows. Representative results of

two independent experiments. **C** – Average values ± SD from the two independent rosetting assays.

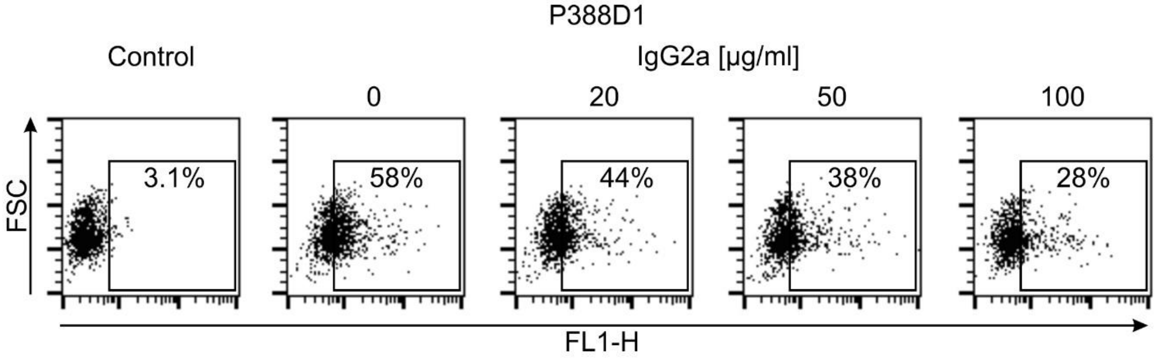

Figure S10 – M18-488 binding in the presence of IgG2a. The panel presents results from one experiment. M18-488 was used at a concentration of 50 µg/ml. The clone #12 of P388D1 was used in the experiment.

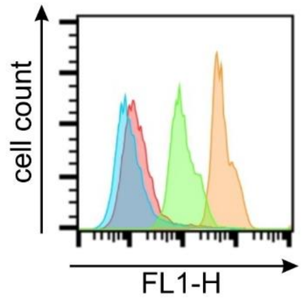

	Pretreatment		Staining	
	isotype Ab	HMβ1-1	anty-CD-29 primary Ab	seconary Ab
	-	+	-	+
	+	-	-	+
	-	+	+	+
	+	-	+	+

Figure S11 – Internalization of ITGB1 upon treatment with HMβ1-1 – an ITGB1-blocking antibody. P388D1 cells were pretreated with 20 μg/ml of HMβ1-1 or matched isotype control for 30 minutes at 37 °C. Then, the cells were chilled on ice and stained with goat polyclonal anti-ITGB1 (AF2074, 2.5 μg/10^6 cells, R&D Systems). The bound goat antibodies were detected with secondary antibody conjugated with Alexa Fluor 488 (donkey anti-goat IgG, 10 μg/ml, Thermo Fisher). The incubations with the polyclonal anti-ITGB1 antibody and with the labelled detection antibody lasted for 30 min and were done on ice or at 4 °C to minimize internalization of plasma membrane. Dead cells were excluded using 7-AAD (2.5 μg/mL). Representative result of three independent experiments.

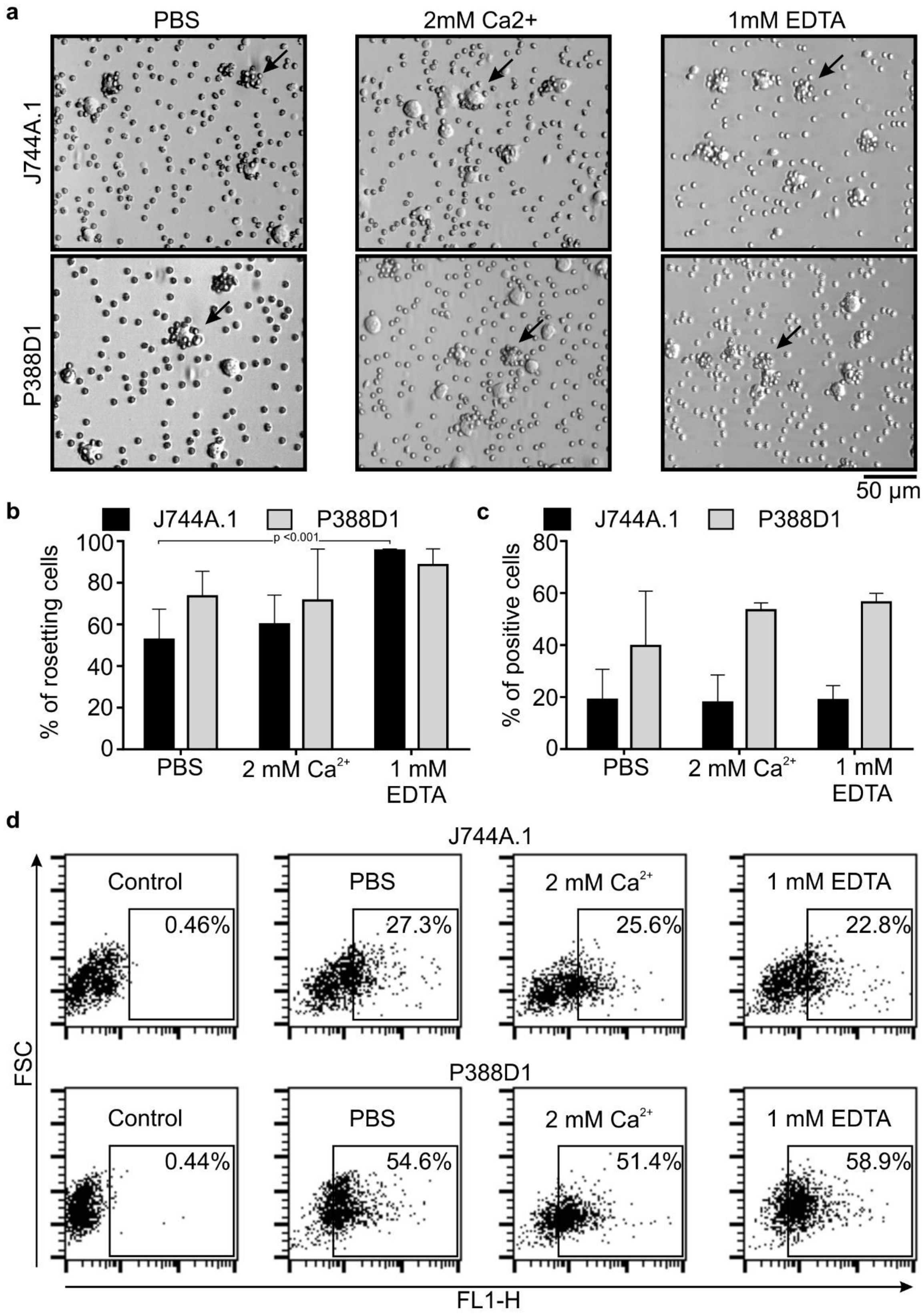

Figure S12 – Mouse IgG3 binding to J774A.1 and P388D1 cells in the presence of calcium ions. **a** and **b** – Rosettting assay. J774A.1 and P388.D1 cells were coated with mIgG3 M18 specific to antigen B of the ABO blood group system. Then, the cells were incubated with

group B erythrocytes. All steps of the assay were done in the presence of 2 mM Ca^{2+} or 1 mM EDTA. **a** – Representative microscope images of two independent experiments are shown. Exemplary rosettes are indicated with arrows. **b** – Average values ± SD from the two independent rosetting experiments. Three different fields were analyzed per sample in each of the experiments. **c** and **d** – Binding of mIgG3 M18-488 to J774A.1 and P388D1 cells in the presence of Ca^{2+} or EDTA. The cells were stained with the labeled antibody and then analyzed using a flow cytometer. **c** – Average values ± SD of two independent experiments. **d** – Representative dot plots from the flow cytometry analysis. **a-d** – The clone #12 of P388D1 was used in these experiments.

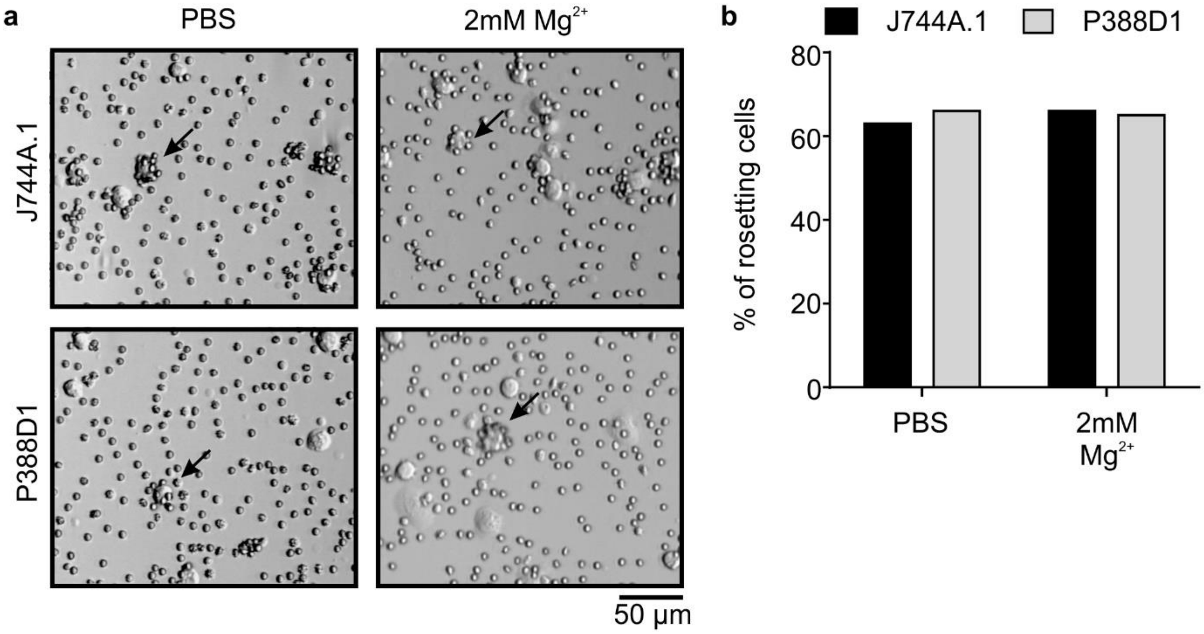

Figure S13 - Mouse IgG3 binding to J774A.1 and P388D1 cells in the presence of magnesium ions. **a** and **b** – Rosetting assay. J774A.1 and P388.D1 cells were coated with mIgG3 M18 specific to antigen B of the ABO blood group system. Then, the cells were incubated with group B erythrocytes. All steps of the assay were done in the presence of 2 mM Mg^{2+}. **a** – Representative microscope images are shown. Exemplary rosettes are indicated with arrows. **b** – Quantification of rosetting cells. Three different fields were analyzed per sample. The experiment was done once. The clone #12 of P388D1 was used in the experiment.

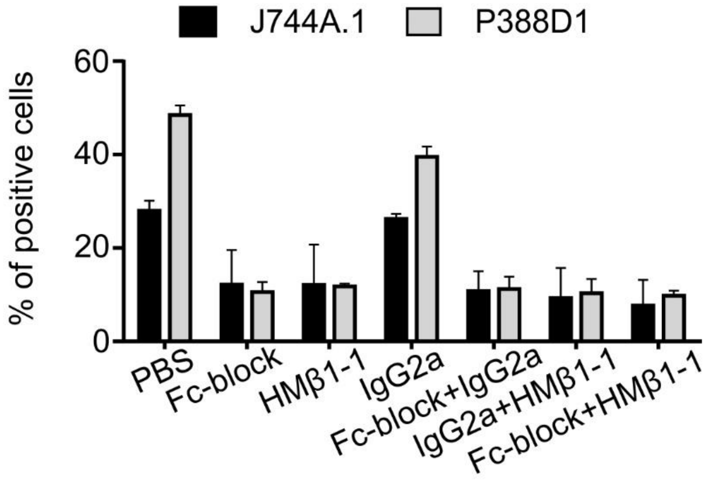

Figure S14 – This figure presents extended bar chart from the Figure 3c. The results obtained for pairs of the blocking antibodies were added here.

Figure S7 - FcγRI

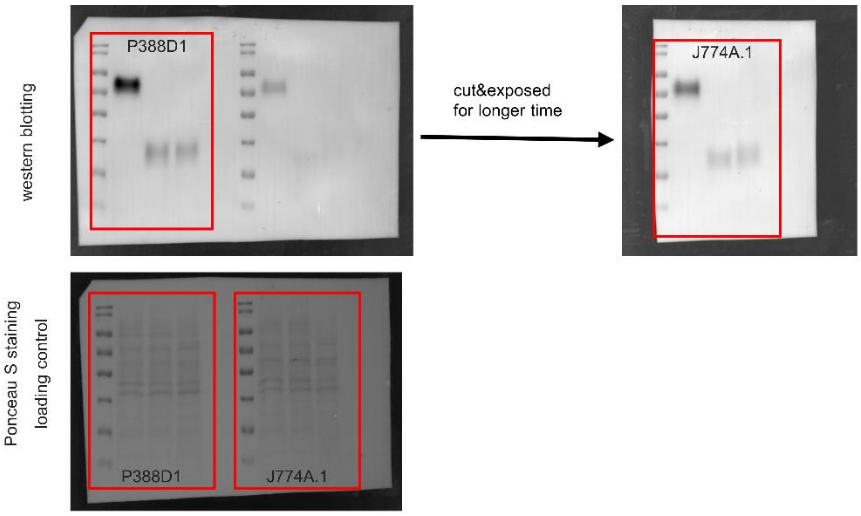

Figure S7 - FcγRII/FcγRIII

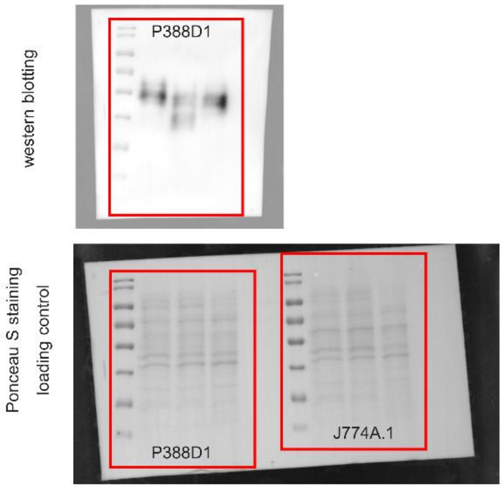

Figure S7 - Itgb1

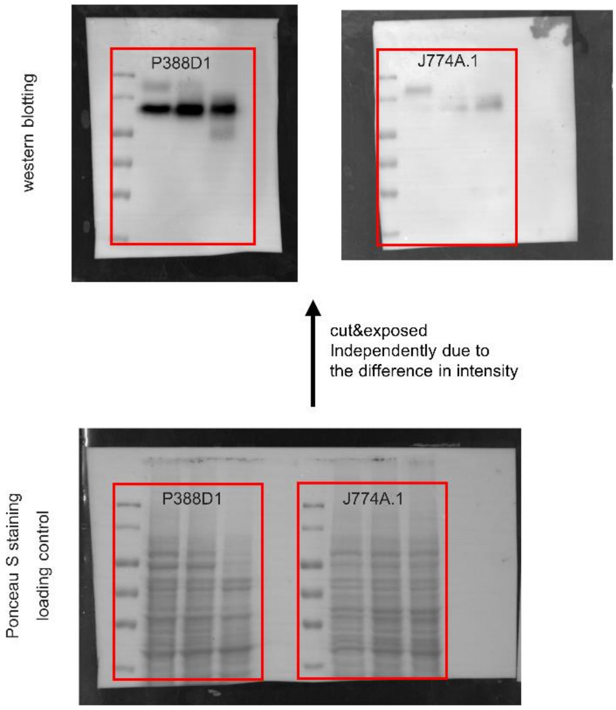

Figure S9 – ADAM17

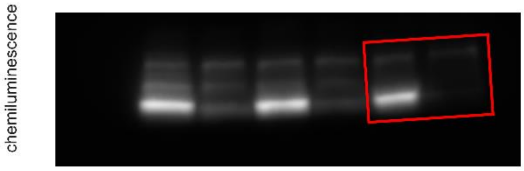

Figure S9 – GAPDH

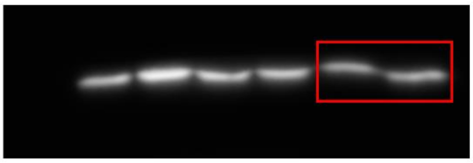

Figure S15 – Uncropped images of blots.

References – Supplementary Figures

[1] J. Vier, M. Groth, M. Sochalska, S. Kirschnek, The anti-apoptotic Bcl-2 family protein A1/Bfl-1 regulates neutrophil survival and homeostasis and is controlled via PI3K and JAK/STAT signaling, Cell Death Dis, 7 (2016) e2103.

[2] L. Cong, F.A. Ran, D. Cox, S. Lin, R. Barretto, N. Habib, P.D. Hsu, X. Wu, W. Jiang, L.A. Marraffini, F. Zhang, Multiplex genome engineering using CRISPR/Cas systems, Science, 339 (2013) 819-823.
